# Treg Inducing Adjuvants for Therapeutic Vaccination Against Chronic Inflammatory Diseases

**DOI:** 10.3389/fimmu.2013.00245

**Published:** 2013-08-20

**Authors:** Chantal Keijzer, Ruurd van der Zee, Willem van Eden, Femke Broere

**Affiliations:** ^1^Immunology, Infectious Diseases and Immunology, Faculty Veterinary Medicine, University Utrecht, Utrecht, Netherlands

**Keywords:** regulatory T-cells, immunologic adjuvants, therapeutic vaccines, Treg inducing adjuvants, autoimmunity

## Abstract

Many existing therapies in autoimmune diseases are based on systemic suppression of inflammation and the observed side effects of these therapies illustrate the pressing need for more specific interventions. Regulatory T-cells (Treg) are pivotal controllers of (auto-aggressive) immune responses and inflammation, and decreased Treg numbers and/or functioning have been associated with autoimmune disease. Therefore, Treg became frequently studied targets for more specific immunotherapy. Especially antigen-specific targeting of Treg would enable local and tailor made interventions, while obviating the negative side effect of general immuno-suppression. Self-antigens that participate in inflammation, irrespective of the etiology of the different autoimmune diseases, are held to be candidate antigens for antigen-specific interventions. Rather than tolerance induction to disease inciting self-antigens, which are frequently unknown, general self-antigens expressed at sites of inflammation would allow targeting of disease independent, but inflammatory-site specific, regulatory mechanisms. Preferably, such self-antigens should be abundantly expressed and up-regulated at the inflammatory-site. In this perspective heat shock proteins (Hsp) have several characteristics that make them highly attractive targets for antigen-specific Treg inducing therapy. The development of an antigen-specific Treg inducing vaccine is a major novel goal in the field of immunotherapy in autoimmune diseases. However, progress is hampered not only by the lack of effective antigens, but also by the fact that other factors such as dose, route, and the presence or absence of an adjuvant, turned out to be critical unknowns, with respect to the effective induction of Treg. In addition, the use of a Treg inducing adjuvant might be required to achieve an effective regulatory response, in the case of ongoing inflammation. Future goals in clinical trials will be the optimization of natural Treg expansion (or the induction of adaptive Treg) without loss of their suppressive function or the concomitant induction of non-regulatory T-cells. Here, we will discuss the potential use of protein/peptide-based vaccines combined with Treg inducing adjuvants for the development of therapeutic vaccines against chronic inflammatory conditions.

## Introduction

Autoimmune diseases are characterized by unwanted responses to self-antigens. Current treatments are symptomatic approaches that induce a generalized immune suppression with all the potential side effects of suppressed immune responses against infectious diseases and cancer. A more specific targeting of the underlying cause of disease would be of great benefit for patients. Development of a therapeutic vaccine that can restore immune tolerance in autoimmunity or other non-infectious chronic inflammatory diseases might induce long-term disease remission with minimal side effects.

In healthy individuals immunological self-tolerance is maintained by several mechanisms amongst others by regulatory T-cells (Treg) (1, 2). Loss of Treg function or frequency can lead to autoimmune diseases like type 1 diabetes (T1D) and rheumatoid arthritis (RA). Although several functional and phenotypical characteristics have been linked to Treg, nowadays Treg are still mostly identified by the expression of CD25 and most Treg express the transcription factor forkheadbox P3 (FoxP3) (3, 4). Treg can suppress effector functions of the immune system with a variety of mechanisms. Upon activation, Treg can produce anti-inflammatory cytokines, consume IL-2, directly lyse effector cells, interrupt other paths of the metabolism of effector T-cells, or interact with antigen presenting cells (APCs) that subsequently down regulate their function, thus potentially spreading the suppressive activity (3). Even though many different mechanisms have been described for their suppressive activity in different models and with potentially different Treg, all pathways have one point in common like most other immune cells, Treg need to be activated to become suppressive. Once activated, Treg can suppress immune responses also to other antigens, which is called “bystander suppression” (5).

Many autoimmune diseases and other chronic inflammatory disorders are characterized by either defective Treg function or reduced frequency of Treg, both leading to a dysfunction of Treg capacity. Targeting Treg with the appropriate antigen-adjuvant combination to enhance the Treg functionality can therefore result in dampening of inflammation and is an attractive route to long-term disease remission.

## Vaccination Against Autoimmunity

Traditionally, vaccination is focused on the prevention of pathology from infectious diseases by the induction of a strong protective immune response against the pathogen. The successful implementation of numerous vaccines against infectious diseases reduced worldwide mortality tremendously over the last decades. However, in the meantime, in the Western World increased incidence of several inflammatory disorders such as allergies and autoimmune diseases, like T1D, multiple sclerosis (MS), and inflammatory bowel disease (IBD) have been observed (6). The exact explanation for this increased prevalence of non-infectious inflammatory diseases in the Western World remains elusive. It has been suggested that the increased hygiene and the subsequent reduced number of infections encountered during childhood contribute to the increase in atopy and autoimmune diseases as stated in the “hygiene hypothesis” originally postulated by Strachan (6).

Originally the hygiene hypotheses focused on a disbalance between Th1 and Th2 responses, however, recent advances in the field of immunological homeostasis revealed an important role for Treg in immune regulation and therefore deregulation.

Rook (7) hypothesized that some specific pathogens play an important role in the adaptation and maturation of our immune system during life. Absence of these so-called “old friends” might contribute to the increased prevalence of non-infectious chronic inflammatory conditions since immune education is incomplete. Examples of such old friends could well be saprophytic mycobacteria, multiple helminths, some gut microbiota, hepatitis A virus, *Helicobacter pylori*, *Salmonella*, *Toxoplasma gondii*, and fermenting lactobacilli. The resolution of post-infection inflammation is regarded to go along with the enforcement of immune homeostasis and the expansion of Treg. It is a fact that the immune system is activated by different pathogen-associated molecular patterns (PAMPs) via pattern recognition receptors (PRRs) and these pathways have been exploited in the development of novel adjuvants over the past decades. Although most adjuvant studies focused on the enhancement of effector responses some immune stimulatory antigens obtained from these old friends can enhance Treg function or frequency.

**Figure 1:**
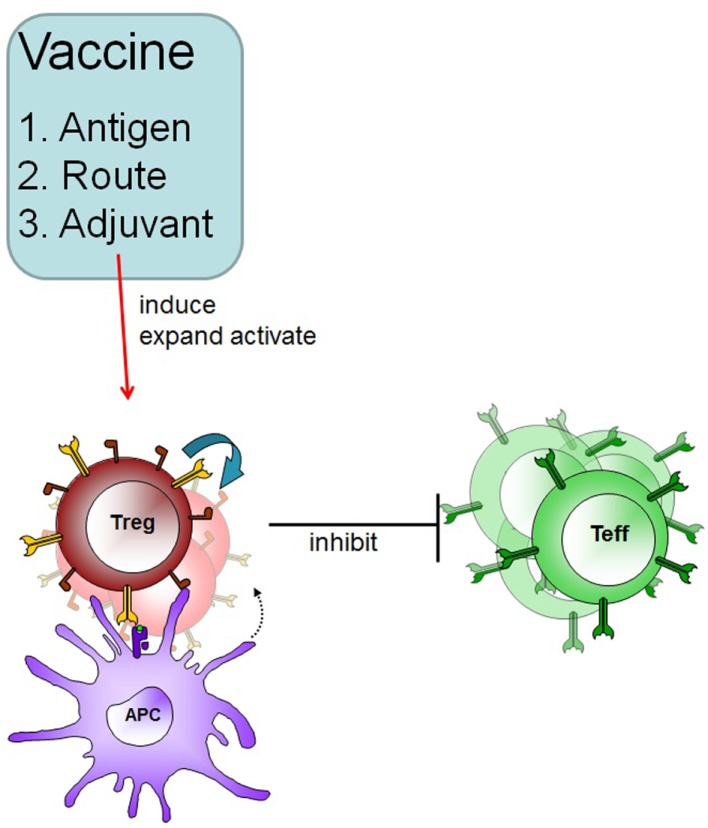
**Therapeutic vaccination in autoimmune disease**. A specific vaccine must induce Treg in an antigen-specific fashion and these Treg can subsequently suppress effector cells in an inflammatory environment. Adjuvants and routes of administration will determine the outcome of therapeutic vaccination.

To develop an antigen-specific immunotherapeutic vaccine for the use in patients with autoimmune disease several points have to be considered (Figure 1).

First the nature of the antigen: in the case of autoimmunity the exact nature of the disease-inducing antigen in most cases remains elusive. Antigen-specific activation of Treg via the T-cell receptor (TCR) is a prerequisite for their functionality (5), therefore we pose the question: which (self) antigens are possible targets?

Secondly, how to induce a Treg response upon vaccination? The majority of vaccines that induce strong adaptive responses are injected parenterally, whereas mucosal application of self-antigens has been shown to induce strong tolerogenic responses against the self-antigen in several animal models of autoimmunity. Translation of so-called mucosal tolerance induction to the clinic has not been successful. It is possible that the use of (novel) adjuvants will cause a breakthrough in this area, either by favoring the induction of Treg upon parenteral vaccination, or by enhancing the mucosal immune response, thereby potentiating the induction of mucosal tolerance in patients with autoimmune diseases. Thus the third aspect that will determine the efficacy of a therapeutic vaccine can be the nature of the adjuvant. Several adjuvants have been described that directly induce adaptive Treg, or indirectly enhance Treg induction via dampening inflammation. For enhancing the specific features of mucosal vaccination we may aim to enhance mucosally induced Treg or to additionally enhance Treg activity of existing Treg via innate receptors. Different potential adjuvants will be discussed in more detail below.

## Antigen-Specific Therapeutic Vaccines; Choice of Antigen

Treg need to be activated before they are functionally suppressive for example via the TCR (5), this directly implies that it is important to know the nature of the desired antigen specificity of Treg to develop an effective vaccine. In the case of autoimmune diseases, autoantigens could possibly serve as targets for such antigen-specific Treg. However, it is questionable whether the typical autoantigens associated with specific autoimmune diseases are sufficiently abundant to serve as targets, especially in cases where the antigen is scarce due to tissue destruction. In addition, in many cases of autoimmune disease epitope spreading plays an important role in the etiology of disease and no single antigen can be defined as the disease-inducing antigen. For a functional Treg mediated suppression it is supposed that only very abundant self-antigens may serve as targets for an effective Treg mediated suppression (4).

Examples of abundant self-antigens are stress proteins. Stress proteins are expressed in every cell and specifically up-regulated at the site of inflammatory reactions. In addition, stress proteins are abundantly present in MHC molecules to be presented to T-cells (8). Recently we have demonstrated that such exposed stress protein fragments may be targeted to Treg. A peptide derived from heat shock protein 70 (Hsp70) was found to have the capacity to induce a very potent Treg response. Following immunization with this Hsp70 peptide responding CD4^+^CD25^+^ spleen lymphocytes had a high capacity to suppress disease in an experimental model of arthritis upon adoptive transfer.

T-cells specific for self-Hsp have been demonstrated by different groups to be beneficial in both autoinflammatory diseases and models for chronic inflammation (9). For example the presence of Hsp60-specific T-cells in juvenile idiopathic arthritis (JIA) patients correlates with a beneficial disease outcome, and has been shown immunoregulatory in RA and juvenile dermatomyositis (JDM). In addition an Hsp-derived peptide, DnaJp1, with good binding capacity to human HLA-DR alleles, not only induced immune deviation in peripheral blood mononuclear cells (PBMC) from patients after oral treatment but also reduced disease severity in responsive patients in a phase II clinical setting (10, 11).

In an experimental skin allograft transplant model in mice, the whole mycobacterial Hsp70 protein was found to induce graft tolerance. Treatment of the graft tissue with Hsp70 prior to transplantation delayed rejection of the graft in a Treg dependent fashion (12).

The first known therapeutic vaccine based on an Hsp-derived peptide is known as DiaPep277. This is an Hsp60-derived peptide that has been shown to induce Hsp-mediated immune balance in T1D (13, 14). This peptide-specific therapeutic approach was extremely successful in the preclinical phase and has shown its potential in clinical trials. Recently in phase III clinical studies where newly diagnosed patients were treated with the Hsp60 peptide for 2 years at quarterly intervals independent of insulin treatment very promising results were obtained. Not only was DiaPep277^®^ -treatment safe and well tolerated. Significant reservation of C-peptide levels was observed, treated patients experienced fewer hypoglycemic events with a significant difference in the rate of decline in the hypoglycemic events/month. In short, Hsp60 peptide treatment preserved beta-cell function and improved clinical outcomes over 2 years in newly diagnosed T1D patients (15).

It is clear that rational development of a therapeutic vaccine is no easy task, not only the selection of a potential vaccine antigen but also the application and adjuvant combination are crucial for the outcome of the immune response. A potentially harmless antigen can induce a serious inflammatory T and B cell response when administered under the wrong conditions.

## Ways to Enhance Treg

### Route of vaccination; mucosal vaccination

**Figure 2:**
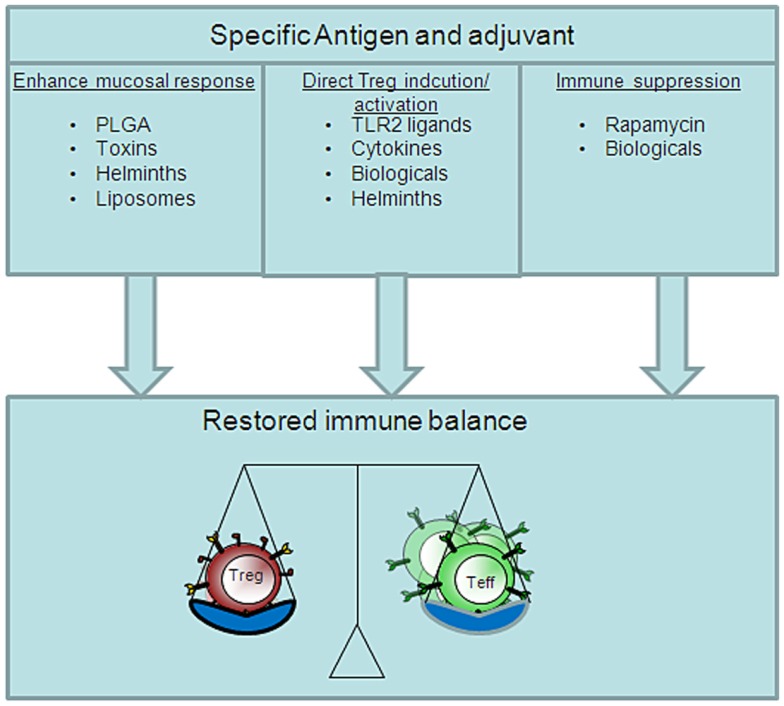
**Distinct pathways of Treg inducing adjuvants**. The restore immune balance in autoimmunity via vaccination depends on several different vaccine characteristics. Therapeutic vaccination can be pursued via a tolerogenic route, adjuvant, or suppression of effector T-cells. The perfect vaccine will most likely contain all these aspects.

Although most infections and environmental allergens are acquired through the mucosal membranes, surprisingly, most vaccines are still delivered via the parenteral route because this route of vaccination seems associated with protective antigen-specific cellular and humoral immune responses. An alternative to conventional multiple injection therapy is the mucosal route of vaccine delivery. Compared to parenteral vaccination one major advantage of the mucosal route of vaccination is the ability to activate both mucosal and systemic immune responses. In addition, vaccination at one mucosal site can induce immunity at peripheral mucosal sites via the common mucosal immune system (16, 17). There are various routes of mucosal vaccination of which the oral and nasal route are most accepted and easily accessible. The immunological outcome of mucosal vaccination depends partially on the antigen dose and the frequency of administration. A low antigen dose frequently applied or a single high antigen dose can both promote the induction of mucosal tolerance, whereas, a single low antigen dose and frequent high antigen application can break mucosal tolerance (18). The vaccine characteristics required depend largely on the immune responses that are desired and can be divided into two categories, pro-inflammatory immune responses against invading pathogens or tumors and tolerance induction to allergens or autoantigens. In general, the induction of antigen-specific Treg after mucosal vaccination is the hallmark for mucosal tolerance induction (19). The forkhead transcription factor, FoxP3, can control both induced and natural Treg cell development and function and these FoxP3^+^ Tregs can secrete anti-inflammatory cytokines such as IL-10 or TGF-β. However, recently, it has been reported that natural or thymus derived Treg but not induced or peripherally derived Treg can convert into Th17 cells after exposure to IL-6 and TGF-β (20). Besides Th1 cells, Th17 cells are major pathogenic effector T-cells in many autoimmune diseases. On the other hand, therapeutic vaccine induced Treg may not be in effective amounts to suppress the pool of activated effector T-cell responses at the site of inflammation. To overcome these problems, co-administration of Treg inducing adjuvants might potentiate the Treg immunoregulatory function (Figure 2).

### Adjuvants to enhance Treg

A flurry of new therapeutic targets in autoimmune diseases has emerged over the past years. However, in the case of autoantigens, co-administration of an adjuvant is often required to potentiate the immunogenicity of the antigen. In addition, adjuvants can be used to reduce the amount of antigen and the number of immunizations needed for protective immunity and to enhance the delivery of antigen to mucosal sites. The type of immune response that adjuvants elicit depends on their specific physicochemical characteristics. Here, we summarize the application of several Treg inducing adjuvants in inflammatory diseases.

#### Cholera toxin B subunit

A first group of potent adjuvants that has been shown to enhance the immune response after mucosal vaccination are the toxin-based adjuvants. The enterotoxin cholera toxin (CT) from *Vibrio cholera* is an example of a potent mucosal adjuvant that can induce systemic and mucosal immune responses. However, the use of enterotoxins as adjuvant should be avoided based on previously reported severe adverse effects attributable to their toxic nature (21, 22). Therefore, to improve safe use of CT, a non-toxic mutant has been developed also known as the B subunits of CT (CTB). The non-toxic recombinant (CTB) has been described to enhance protective immune responses against influenza virus (23, 24). However, combined with different antigens it can also enhance Treg responses in several autoimmune disorders. For example, B cells that were pretreated with antigen conjugated to CTB *in vitro* produced IL-10 and induced a B cell dependent increase in the number of FoxP3^+^ Treg upon adoptive transfer, resulting in protection against experimental autoimmune encephalomyelitis (EAE) both prior to and after disease induction (25). In a more direct vaccination approach Ploix et al. showed that oral administration of CTB-insulin conjugates protected NOD mice from autoimmune diabetes. The delayed onset of diabetes relied on a shift from a Th1 to a Th2 profile in pancreatic lymph nodes and an increase in TGF-β in the mesenteric lymph nodes (MLN) and the induction of antigen-specific CD4^+^ Treg in the area of the mucosal barrier and inflamed islets (26). A low-density lipoprotein peptide (3136–3155) of apolipoprotein B-100 fused to CTB, when intranasally applied, induced IL-10 producing Tr1 cells that reduced atherosclerosis by suppressing the activity of antigen-specific effector T-cells (27).

Recently, ADP-ribosylation was shown to control the outcome of the CD4^+^ immune response after mucosal antigen exposure with OVA inserted into the CT-derived CTA1-DD monomeric fusion protein that consist of the enzymatically active CT A1 subunit (CTA1) combined with two Ig-binding domains, DD, of staphylococcal protein A (28). Here, mucosal tolerance was associated with the induction of IL-10 producing CD4^+^CD25^−^FoxP3^−^Type I Treg (Tr1) cells (29).

Overall, CTB has been shown to modulate Treg. However, it remains difficult to categorize this adjuvant, because of its ability to break or enhance mucosal tolerance induction, which is influenced by the nature of the antigen that is co-administered as well as the dose, route, and time of application. Future research to improve our understanding of the immunoregulatory mechanisms of CTB is required to predict the direction of the immune response.

#### TLR2 ligands

During natural infections many different Toll-like receptors (TLR) are activated and TLR mediated activation of APCs can contribute to the induction of efficient adaptive effector responses. Different TLR2 agonists can induce distinct cytokines pattern and signaling in APCs and differentially skew T helper cell responses *in vivo*. For example, in allergic airway inflammation, a Th2 associated inflammatory airway disease, the TLR2 agonist Pam3CSK4 enhanced the production of IL-12 by DCs *in vitro* and *in vivo* therapy attenuated established asthma in mice by shifting the antigen-specific T cell response toward a Th1 response (30). On the other hand, after intranasal or intraperitoneal immunization, the TLR2 ligand diacylated lipopeptide FSL-1 enhanced the generation of Th2-type IgG1 antibodies compared to the Th1-type IgG2a antibodies (31). However, specific TLR2 activation might preferentially enhance the induction and activation of Treg (32). The fact that TLR2 signaling can induce such a diverse range of responses is most likely due to the fact that TLR is a transmembrane signaling protein that can form heterodimers with co-receptors such as TLR1, TLR6, and Dectin-1 and ligand and receptor characteristics will determine the outcome of the response. Signaling through the TLR2/TLR1 heterodimer was shown important in the induction of protective mucosal Th17 immune responses against infection (33), whereas intranasal treatment with the synthetic TLR2/TLR1 agonist Pam3Cys induced an expansion of the Treg cell population in the lungs in a mouse model of allergic asthma (34), TLR2/TLR6 receptor ligation by *Yersinia pestis* virulence factor LcrV and Yeast Zymosan, a ligand for TLR2/Dectin-1 both promoted tolerogenic dendritic cells (DCs), and the induction of Treg responses (35, 36).

Moreover, the Hsp60 peptide, Diapep277 that was shown to be a protective peptide in the NOD diabetes mouse model by inducing antigen-specific Tregs also enhanced CD4^+^CD25^+^ Treg cell function via innate TLR2 signaling (14). In addition, a recently conducted phase 3 multicenter trial showed that the p277 trial had reached its primary and secondary clinical endpoints in T1D patients (37). These data have shown the unique potential of Treg inducing vaccination in autoimmunity and a role for TLR2 mediated immune activation.

TLR2 ligands can be applied to enhance the induction of Treg *in vivo* and might be potential adjuvants in the treatment of chronic inflammatory disease. Caution is needed when selecting appropriate TLR2 adjuvants for Treg induction, because some ligands for TLR2/TLR1 signaling such as Pam3Cys have been shown to skew Treg toward a Th17 phenotype and might therefore increase the risk of developing autoimmune disease (38). Both the route of TLR2 adjuvant application and TLR2 receptor ligation can significantly affect the immunological outcome of vaccination and need to be considered when selecting TLR2 ligands as vaccine adjuvant.

#### Helminth-derived excretory/secretory immunoregulatory products

The “Hygiene Hypothesis” states that a lack of early childhood exposure to infectious agents, microorganisms, and parasites increases susceptibility to allergic diseases, particularly in the developed world. Recently, the hygiene hypothesis has been extended to also incorporate inflammatory and autoimmune diseases in general (39). There is substantial evidence from animal models of human disease that infection with helminths can suppress ongoing inflammation in autoimmune diseases such as arthritis (40), IBD (41), and EAE (42) by modulation of the immune response.

The main disadvantage of using the whole parasite to treat disease in general is the fact that infection with helminths can cause severe pathology and permanent damage to the host. Studies on the mechanism of immune modulation by helminths showed that helminth-derived excretory/secretory (ES) products including proteins, lipids, and glycoconjugates can suppress DC maturation (43, 44) and modulate DC functionality and induce CD4^+^CD25^+^FoxP3^+^ Treg *in vitro* (45) and *in vivo* (46). For example, a *Schistosoma*-derived lysophosphatidylserine molecule that specifically activated TLR2, affected DC function and stimulated the development of IL-10-producing Treg (47).

In addition, a single ES molecule named ES-62 derived from the filarial nematode *Acanthocheilonema viteae* can suppress collagen induced arthritis (CIA) by targeting IL-17 producing cells (48). The main advantage of using helminth-derived ES products is that a regulatory response can be induced that could dampen chronic inflammation in the absence of infection *per se*, and, increase safe application and reduce the chance of therapy associated side effects. The fact that helminth-derived ES products can induce immunoregulatory immune responses in a variety of animal models of human disease suggests that helminth-derived ES products act independently of the actual disease-inducing trigger. Since helminth-derived ES products can induce Treg, they can be used as potential vaccine adjuvants for successful therapeutic intervention and might enable local activation of Treg at the site of inflammation.

#### Particulate antigen delivery systems

An advantage of vaccine delivery particles compared to soluble adjuvant is that the target antigen can be encapsulated by the particle to protect it from enzymatic degradation. Vaccine delivery particles allow large-scale production and long-term storage. Moreover, particles can be easily modified to enhance their mucoadhesiveness to epithelial cells that line the mucosal sites or specific ligands can be incorporated that enhance tolerogenic immune responses or ensure uptake by APCs.

##### Polylactic-co-glycolic-acid

Polylactic-co-glycolic-acid particles are available as non-toxic biodegradable vaccine delivery systems with promise for both mucosal and non-mucosal application. Recently, PLGA particles were demonstrated to enhance retinaldehyde dehydrogenase enzyme activity in CD11c^+^ DCs that led to and enhanced induction of CD4^+^FoxP3^+^ T-cells, *in vitro*, via a retinoic acid, and TGF-β dependent mechanism (49). Observations that PLG microparticles can prolong the expression of the major histocompatibility complex (MHC) class I and the MHC class II molecules on the cell surface of DCs, while they do not significantly enhance maturation of DCs (50), suggest that PLGA particles modulate DC phenotype and function to enable them to enhance tolerogenic immune responses.

Oral tolerance induction by a single dose of 40 μg of type II collagen (CII)-loaded PLGA particles reduced severity of CIA, lowered T-cell responses, and resulted in a higher expression of TGF-β mRNA in the Peyer’s patches and this implies that PLGA particles can be applied to enhance the induction of oral tolerance to the target antigen (51).

Nasal application of PLGA particles in mice enhanced mucosal tolerance via the induction of CD4^+^FoxP3^+^ Treg in the nose draining lymph nodes. Combined with an immunosuppressive Hsp70 peptide, PLGA particles suppressed the onset of disease and arthritis symptoms in a proteoglycan induced arthritis mouse model (52).

The uptake of microparticles (polystyrene or PLGA) bearing encephalitogenic peptides (PLP_139–151_ epitope) by marginal zone macrophages that express the scavenger receptor macrophage receptor with collagenous structure (MARCO) ameliorated EAE by the activation of Treg that inactivated pathogenic effector T-cell responses (53).

These studies show the potential of PLGA particles to be applied as enhancers of T-cell mediated tolerance in autoimmunity.

##### Liposomes

Next to PLGA particles also other particulate systems might aid in the induction of tolerance. Liposomes are vesicles composed of one or more phospholipid membranes that can deliver a broad range of molecules (54). Liposomes can be mixed, coated, or loaded with a variety of antigen including protein, peptide, DNA-complexes, and even whole inactivated virus vaccine and such tailor made liposomes can be utilized to steer the immune response after vaccination. Liposomes loaded with OVA and NF-κB inhibitor induced antigen-specific FoxP3^+^ Treg and methylated BSA and NF-κB inhibitor loaded liposome treatment reduced the severity of antigen-induced arthritis in mice (55). Next to NF-kB inhibitors other immunosuppressants can be encapsulated, such as rapamycin analogs (see below). Moreover, addition of specific targeting molecules to enhance antigen uptake by tolerogenic DCs (e.g., via DEC205) might also enhance the tolerogenic capacity of liposomes (56).

In conclusion, liposomes can be used as antigen delivery systems in a variety of diseases and due to the fact that also other immune modulating compounds can be co-packaged liposome content can be adjusted to induce Tregs.

### Immunosuppressant drugs

When considering a therapeutic vaccination in the case of autoimmune disease in most cases such a novel antigen-specific therapeutic approach will be applied in combination with a form of general immune suppression as most current therapies comprise. However to restore immune balance the immunosuppressant drug might be considered potential Treg inducing adjuvants A suggested common mechanism whereby immunosuppressant’s such as dexamethasone, vitamin D3 analogs, and rapamycin analogs can enhance Treg induction is via the induction of immature or tolerogenic DCs that express lower levels of MHC class II and co-stimulatory molecules (CD40, CD80, and CD86) and do not produce pro-inflammatory cytokines and promote Treg differentiation upon antigen presentation (57). Here, we describe some immunosuppressant drugs, that can potentially act as Treg inducing adjuvant.

#### Rapamycin and analogs

Rapamycin and its analogs everolimus, fingolimod, and tacrolimus are immunosuppressive drugs and target mTOR. Rapamycin inhibits the protein kinase activity of mTOR that promotes cell growth and metabolism (58). In the clinic, rapamycin is often used as a drug to suppress CD4+ “memory” T-cell responses to prevent allograft rejection (59). However, rapamycin can also efficiently convert naïve T-cells into adaptive Treg and re-establish long-term immune self-tolerance in a variety of autoimmune diseases. Rapamycin promoted the expansion of functional CD4^+^CD25^+^FOXP3^+^ Treg in both healthy individuals and in T1D patients (60, 61). Moreover, rapamycin monotherapy already affected human CD4^+^CD25^+^FOXP3^+^ Treg function directly *in vivo* as nTreg isolated from T1D patients under rapamycin treatment had an increased capability to suppress proliferation of CD4^+^CD25^−^ effector T-cells compared with that before treatment, without inducing alterations in the frequency of circulating nTreg and proliferation and cytokine production (62).

In an animal model for EAE rapamycin was found to increase the percentage of CD4^+^CD25^+^FoxP3^+^ Treg and reduced the clinical signs (63–65).

Most studies directly studied the immunosuppressive effects of rapamycin on the heterologous Treg population, however, more recently rapamycin has been shown to be a functional adjuvant that may induce and expand antigen-specific Treg. Wu et al. showed that mice immunized with OVA with either rapamycin or fingolimod had significantly higher percentage and cell number of antigen-specific CD4^+^CD25^+^FoxP3^+^ Treg compared to mice that received protein immunization alone. These data show the potential of rapamycin and its analog fingolimod as potential Treg inducing adjuvants. This effect could solely be attributed to the immunosuppressive function of rapamycin and fingolimod as mice immunized with OVA and treated with other immunosuppressive drugs such as cyclosporin A, mycophenolate mofetil, leflunomide, or cyclophosphamide showed decreased levels of OVA-specific CD4^+^CD25^+^FoxP3^+^ Treg in the draining lymph nodes and spleen (66).

The rapamycin analog everolimus can convert naïve T-cells into FoxP3^+^ Treg *in vivo* by interfering with T-cell costimulation and by reducing proliferation. A combination therapy of everolimus and IL-2/IL-2ab complexes could even further enhance the number of IL-10 secreting anergic CD25^+^FoxP3^−^ T-cells *in vivo*, most likely by enhancing expansion of induced Treg in an IL-2 dependent fashion (67). For tacrolimus it was shown that the immunomodulatory action functioned via blocking of DC maturation after it enhanced the number of tolerogenic IL-10 expressing CD11c^+^ DCs. A combination of the p2MOG35 and the adjuvant tacrolimus enhanced the induction of antigen-specific Treg that infiltrated the spinal cord and protected mice from EAE. Protection coincided with decreased expression of IFN-γ and reduced numbers of Th17 cells (68). Although the exact mode of action might differ between rapamycin ramaycin and the different analogs the studies described show that a combination of the immunosuppressive drugs can be used as an immune modulating a immune modulating adjuvant for therapeutic vaccines.

#### Vitamin D3 analogs

Another potential adjuvant that induces tolerogenic DCs is 1α,25-Dihydroxyvitamin D_3_ (1,25(OH)_2_D_3_). The potent inhibition of NF-κB expression leads to tolerogenic DCs with increased potential to induce Treg. A combination of antigen-specific immunotherapy with 1,25(OH)_2_D_3_ in a murine model for asthma enhanced the suppressive effect of therapy in an IL-10 and TGF-β dependent fashion (69). Moreover Treg inducing capacity of vitamin D3 analogs has been shown after topical application of calcipotriol. Calcipotriol treatment reduced Langerhans cell maturation thereby promoting the expansion of antigen-specific Treg and reduced antigen-specific priming of effector CD8^+^ T-cells in an OVA-induced model of skin hypersensitivity (70). The data showed that interfering with DC maturation and induction of tolerogenic DCs favors the induction of Treg upon antigen presentation, however, this is not unique to vitamin D3 analogs and compounds with a comparable mode of action might also be potent adjuvants for the induction of Treg.

#### Retinoic acid

The vitamin A metabolite retinoic acid plays a role in the induction of mucosal tolerance. In more detail, retinoic acid produced by mucosal DCs acts on naïve T- and B-lymphocytes and induces the expression of mucosal homing receptors α4β7-integrin and CCR9. The function of retinoic acid depends largely on the microenvironment especially the cytokine milieu. For example, retinoic acid in the presence of TGF-β mediates the conversion of naïve T-cells into FoxP3 expressing Treg, while retinoic acid alone at high concentration inhibits the differentiation of Th17 cells (71, 72).

In mice with CIA all-trans retinoic acid (ATRA) directly increased the percentages of total FoxP3^+^ Treg cells in the spleens and reduced the expression of IL-17 in the arthritic joints and Th17 cells in the spleens of ATRA treated mice. ATRA treatment down-regulated the expression of RANKL a key osteoclastogenic molecule expressed in CD4^+^ T-cells and fibroblast-like synoviocytes and osteoclast formation in arthritis joints was reduced (73).

All-trans retinoic acid treatment inhibited diabetes in NOD mice with established insulitis by the expansion of Treg cells that suppressed IFN-γ-producing CD4^+^ and CD8^+^ T-cells, without affecting Th17 cells or IL-4 producing cells. In addition, depletion of CD4**^+^**CD25**^+^** Treg impaired the inhibitory effect of ATRA on islet-infiltrating CD8^+^ T-cells and blocked its protective effect on diabetes (74). Moreover, when CD8^+^ T-cells were exposed to ATRA and TGF-β these cells differentiated into CD8^+^Foxp3^+^ Treg *ex vivo*. These CD8^+^ antigen-specific Treg suppressed proliferation of diabetogenic T-cells isolated from NOD mice *in vitro* and could prevent the onset of diabetes in NOD-CSID mice *in vivo* (75). Most studies on the role of retinoic acid describe the induction of heterologous Treg, however, it can be hypothesized that when combined with a specific antigen in a vaccine, antigen-specific Treg will be induced *in vivo*.

To summarize, different immunosuppressive drugs can promote the generation of Treg both *in vitro* and *in vivo*. Since immune suppressive treatment is the current therapy in several autoimmune diseases to suppress effector responses, it can be speculated that these drugs can be effective as Treg inducing adjuvant as part of a therapeutic vaccine for chronic inflammatory disorders.

### Chemical compounds

In general, most adjuvants currently clinically applied in vaccines are chemicals such as MF59, Aluminum hydroxide (Alum), and adjuvant systems including AS04, which is a combination of alum and the TLR4 agonist MPL. These adjuvants are mostly described for their induction of effector responses and protective immunity in infectious diseases such as influenza (76) or anti-cancer immunity (77). However, in some cases also regulatory actions attributed to these adjuvants as described below.

#### Alum

Alum is the most widely used vaccine adjuvant, but its mechanism of action remains largely unknown, therefore it is not surprising that even immunomodulatory actions have been described for a well-known adjuvant as alum. Hjorth et al. recently studied the immunomodulatory effect of alum-formulated glutamic acid decarboxylase 65 (GAD-Alum) in autoimmune T1D patients. PBMCs were isolated from blood samples of GAD-alum injected patients, and restimulated by GAD65. The treatment induced GAD65-specific CD4^+^CD25^high^FOXP3^+^ cells in recent-onset T1D children and adolescents (78). In addition, in alum-treated ApoE^−/−^mice the protective effect of alum relied on an increased percentage of CD4^+^CD25^+^FoxP3^+^ Treg most likely activated by tolerogenic APCs that presented oxidized LDL antigens, while having a down-regulated expression of CD28 and ICOS activation markers (79). These studies show that an adjuvant that has been widely used to induce effector responses can induce Treg under specific circumstances. However, the exact requirements for alum to function as a Treg inducing adjuvant are unclear, more research is needed to unravel this.

#### Incomplete Freund’s Adjuvant

Another adjuvant that has been used widely to induce effector responses in different animal models at least is incomplete Freund’s adjuvant (IFA). NOD mice with prediabetic stages of diabetes can be protected from diabetes after immunization with the 9–23 amino acid region of the insulin B chain (B:9–23) in IFA. A single B:9–23/IFA immunization increased Treg numbers that required IFN-γ and IL-10. CD4+CD25+ and to a lesser extent IFN-γ-producing cells from mice protected by B:9–23/IFA induced tolerance upon transfer into new NOD animals and protection from disease coincided with reduced numbers of diabetogenic NRP-V7+CD8+ T-cells (80).

In this setup both the antigen and the prophylactic timing of the vaccination are likely to contribute to the protective effect. However, whether IFA can also function as a Treg inducing adjuvant in a therapeutic setting needs to be addressed in future studies.

### Monoclonal antibody therapy

Monoclonal antibodies (mAb) that negatively regulate T-cell function can be used as immunosuppressant agents. Antibodies that target CD3 molecules modulate the CD3/TCR complex and are potent immunosuppressive agents. Since the C-terminal domain of the heavy immunoglobulin chain (Fc) of the anti-CD3 antibodies can interact with Fc receptors or C1q molecules, anti-CD3-specific antibodies exhibit toxic mitogenic properties (81). However, non-mitogenic antibodies are available also known as non-Fc-binding anti-CD3 F(ab′)_2_ that can restore self-tolerance in non-diabetic (NOD) mice via the induction of TGF-β producing CD4^+^CD25^+^ Treg cells (82). Combination therapy of systemic anti-CD3 and intranasal human proinsulin II B24-C36 peptide induced antigen-specific Treg that reversed recent-onset diabetes in mice. *In vivo* expanded Treg that produced IL-10, TGF-β, and IL-4 suppressed auto-aggressive CD8^+^ T-cell responses upon adoptive transfer to recent-onset diabetic recipient mice (83).

In LDLR^−/−^ C57CL/6 mice, anti-CD3 antibody treatment reduced plaque development when administered before a high-cholesterol diet and markedly decreased lesion progression in mice with already established atherosclerosis. The anti-atherosclerotic effect was associated with increased TGF-β secretion by *ex vivo* ConA stimulated lymph node cells and FoxP3 expression in spleens of anti-CD3 treated mice (84). Cytotoxic T lymphocyte-associated antigen 4 (CTLA-4) is a negative regulator of T-cell function. CTLA-4-Ig (a fusion protein composed of the Fc region of immunoglobulin IgG1 fused to the extracellular domain of CTLA-4) also known as Abatacept, is an immunosuppressive agent effective in the treatment of RA that blocks the B7/CD28 co-stimulatory interaction and inhibits effector T-cell proliferation. *In vitro*, CTLA-4-Ig treatment in combination with TCR ligation converted naive CD4^+^CD25^-^ T-cells into CD4^+^CD25^+^FoxP3^+^ Treg in an APC-dependent but TGF-β signaling independent manner. *In vivo*, systemic administration of CTLA-4**-**Ig increases the percentage of CD4^+^CD25hiFoxp3^+^ cells within mixed lymphocyte reaction-induced murine lymph nodes (85).

CTLA-4-Ig treatment modified CD11c^+^ DCs from CIA mice into tolerogenic DCs that upon adoptive transfer to recipient mice with induced CIA increased the CD4^+^CD25^+^FoxP3^+^ Treg population in joint and spleen and suppressed IL-17^+^CD4^+^ T-cells (86). Anti-CTLA-4 mAb treatment induced an increased proliferation rate of IL-10 producing ICOS^high^FoxP3^+^CD4^+^T-cells in the MLN and colon, inhibited Th1 memory responses, and ameliorated TNBS-induced colitis in mice (87).

To summarize, monoclonal antibodies can be used to negatively regulate T-cell effector responses directly or via the induction of tolerogenic DCs and Treg.

### Combination therapy

Chronic inflammatory diseases in general are complex disorders where multiplicities of pathogenic elements have a combined contribution to the disease process. Since several immune functions, rather than individual pathways should perhaps be targeted, combination therapies might offer new opportunities to overcome obstacles seen for single approach therapies. The aim of combination therapy would be to dampen pro-inflammatory effector responses at the site of inflammation with a disease modifying biological agent followed by the application of a disease-specific therapeutic agent to increase antigen-specific Treg responses. Roord et al. showed that in rats with adjuvant arthritis, the combination of an arthritogenic peptide (Hsp60 p. 180–188) with one-third of the regular dose of etanercept led to significant disease improvement and regulatory immune deviation (88). Thus, combination therapy offers several advantages over monotherapy, besides increasing the suppressive activity of disease-specific Treg, it most likely will also be effective with a lower dose usage of the immunosuppressive agent thereby reducing the risk of developing therapy-related adverse side effects.

## Conclusion

Here, we have reviewed a variety of adjuvants used to induce Treg that have great potential for future vaccine development in chronic inflammatory autoimmune diseases. Autoimmune diseases are characterized by unwanted responses to self-antigens that are often unknown. This complicates the development of an antigen-specific Treg inducing therapeutic vaccine. Alternatively, Hsp are abundantly up-regulated under conditions of stress, and in that respect, Hsp protein/peptide might induce Hsp-specific Treg that can suppress antigen-specific effector cell responses at the site of inflammation. These immunoregulatory antigens most likely need to be combined with a Treg inducing adjuvant to achieve an effective regulatory response, in the case of ongoing inflammation. Thus, the ideal therapeutic vaccine for chronic inflammatory diseases will require a combination of several approaches, including “mucosal” application of an immunoregulatory antigen, the use of a Treg inducing adjuvant and the ability to suppress antigen-specific effector cells.

## Conflict of Interest Statement

The authors declare that the research was conducted in the absence of any commercial or financial relationships that could be construed as a potential conflict of interest.
